# A coastal hospitality sector database for vulnerability assessments in Veneto and Emilia-Romagna, Italy

**DOI:** 10.1016/j.dib.2025.111921

**Published:** 2025-07-22

**Authors:** Vilane Gonçalves Sales

**Affiliations:** a Department of Environmental Sciences, University of Venice, Venice, Italy; b Fondazione Centro Euro-Mediterraneosui Cambiamenti Climatici (Fondazione CMCC), Venice, Italy

**Keywords:** Coastal tourism, Spatial database, Climate vulnerability, Sea-level rise, Hospitality infrastructure, Geospatial analysis, Environmental certification, Disaster risk management

## Abstract

This article presents a comprehensive geospatial database documenting the coastal hospitality sector across Veneto and Emilia-Romagna regions in Northern Italy. The dataset integrates information on 5030 accommodation establishments and 4992 service facilities, providing detailed spatial and attribute data essential for vulnerability assessments in the context of climate change and sea-level rise projections. Data collection employed a multi-source approach combining web scraping, API queries from Google Places, and crowdsourced repositories (Eubucco, Overture Maps), with meticulous validation protocols ensuring robust data quality. Each record includes precise geolocation, structural characteristics, proximity to coastal features, elevation metrics derived from the TINITALY digital terrain model, sea-level rise projections based on IPCC-AR5 ensemble models, sustainability certifications, and amenity information. The dataset facilitates spatial analysis of hospitality infrastructure vulnerability to coastal hazards, including sea-level rise, erosion, and storm surges. This comprehensive coverage of Northern Adriatic coastal tourism infrastructure offers significant potential for multi-disciplinary applications, including climate adaptation planning, tourism management, and disaster risk reduction for one of Europe's most economically significant coastal tourism regions.

Specifications Table

**Table:** 

Subject	Social Sciences
Specific subject area	Coastal geography with focus on hospitality infrastructure vulnerability to sea-level rise and environmental hazards
Type of data	Table Raw, Analysed, Filtered, Processed
Data collection	Data were collected through a multi-source approach integrating web scraping, API queries, and crowdsourced repositories. Web extraction utilized Python BeautifulSoup and Selenium with exponential backoff algorithms for hospitality establishments, while geospatial metrics were derived from the TINITALY digital terrain model with 10 m resolution. Google Places API provided primary establishment data, supplemented by Eubucco [1] and Overture Maps [2] for building characteristics. Sea-level rise projections were derived from IPCC-AR5 2015 ensemble (Church et al., 2013) under RCP4.5 scenarios. Spatial data integration employed geographical location as the primary matching criterion through QGIS and custom Python scripts. Quality assurance included automated geometric validation, topological assessment, spatial outlier detection, and cross-source attribute consistency evaluation, with 30 % of entries undergoing manual expert review for attribute verification.
Data source location	Institution: University of Venice City/Town/Region: Coastal zones of Veneto and Emilia-Romagna regions Country: Italy Latitude and longitude bounds: 43.7°−45.7°N, 12.2°−13.8°E
Data accessibility	Repository name: Zenodo Data identification number: 10.5281/zenodo.14929876 Direct URL to data: https://zenodo.org/records/14929876
Related research article	None – Submitted work to Journal of Coastal Conservation.

## 1 Value of the Data

• This dataset provides unprecedented spatial detail on the hospitality sector infrastructure across two major Italian coastal tourism regions, offering comprehensive coverage of both accommodation establishments and complementary service facilities. The integration of physical vulnerability metrics with business characteristics creates a unique resource for interdisciplinary coastal management research.
• Researchers can utilize these data to develop and validate predictive models of coastal tourism vulnerability, quantify potential economic losses from sea-level rise and storm surges, and evaluate the effectiveness of adaptation strategies. The dataset's structure enables spatial overlay with additional hazard layers for comprehensive multi-risk assessments.
• The methodological framework employed in data collection and validation can be replicated in other coastal tourism destinations globally, facilitating comparative vulnerability assessments and supporting standardized monitoring approaches for sustainable tourism development under changing climate conditions.
• Policy makers and tourism stakeholders can leverage this dataset to prioritize investments in infrastructure protection, develop evidence-based adaptation strategies, and implement targeted resilience-building measures that account for the specific vulnerabilities of different establishment types and locations.
• Urban planners and emergency management agencies will benefit from the detailed spatial resolution of the database, enabling precise identification of high-risk hospitality clusters and supporting the development of evacuation planning and disaster response strategies specifically tailored to the tourism sector.

## 2 Background

Coastal regions of Veneto and Emilia-Romagna in Northern Italy constitute significant economic assets through their hospitality sector, while simultaneously facing substantial vulnerability to climate change impacts. These areas are experiencing increasing threats from sea-level rise (projected to reach up to 1 m by 2100), coastal erosion, and storm surge intensification, compounded by land subsidence and extensive urbanization in low-lying areas [[3], [4], [5]]. The sustainability of the hospitality infrastructure depends fundamentally on coastal environmental stability, with vulnerability manifesting through three interconnected dimensions: dependence on natural and cultural assets, physical exposure of built infrastructure, and economic viability linked to destination attractiveness [6,7].

Previous vulnerability assessments in coastal hospitality contexts have predominantly employed isolated analytical approaches that fail to capture the multidimensional nature of climate-related risks. Such methodologies typically focus on singular aspects (e.g., physical exposure or asset vulnerability) while neglecting their critical interactions, and regional-scale analyses frequently obscure fine-grained spatial heterogeneities in exposure and vulnerability patterns [8,9]. This database addresses these methodological limitations by providing comprehensive, high-resolution spatial information on hospitality infrastructure across coastal Veneto and Emilia-Romagna. The resulting dataset enables sophisticated vulnerability assessments that integrate physical hazards, socio-economic factors, and spatial exposure dimensions through a unified analytical framework, thereby supporting evidence-based climate adaptation planning for this economically vital coastal tourism region.

## 3 Data Description

The dataset comprises two primary files in CSV format, providing comprehensive coverage of hospitality establishments across the coastal regions of Veneto and Emilia-Romagna: coastal_hospitality_accommodations.csv (689 KB): Contains detailed information on 5030 accommodation establishments, including hotels, holiday rentals, bed and breakfasts, camping facilities, and guesthouses. coastal_hospitality_services.csv (673 KB): Documents 4992 complementary service facilities, including restaurants, shops, cultural venues, entertainment establishments, and seaside facilities.

Additionally, two supplementary files provide essential metadata for categorical variables: metadata_coastal_hospitality_accommodations.csv (1 KB): Contains encoding specifications for categorical variables within the accommodations dataset. metadata_coastal_hospitality_services.csv (1 KB): Provides encoding specifications for categorical variables within the services dataset.

The encoding schemes facilitate computational analysis while preserving the categorical nature of these variables. Missing or unknown values are consistently coded as −1 across all categorical variables and NA across all numerical variables, enabling systematic handling of data gaps during analysis.

**Fig. 1:**
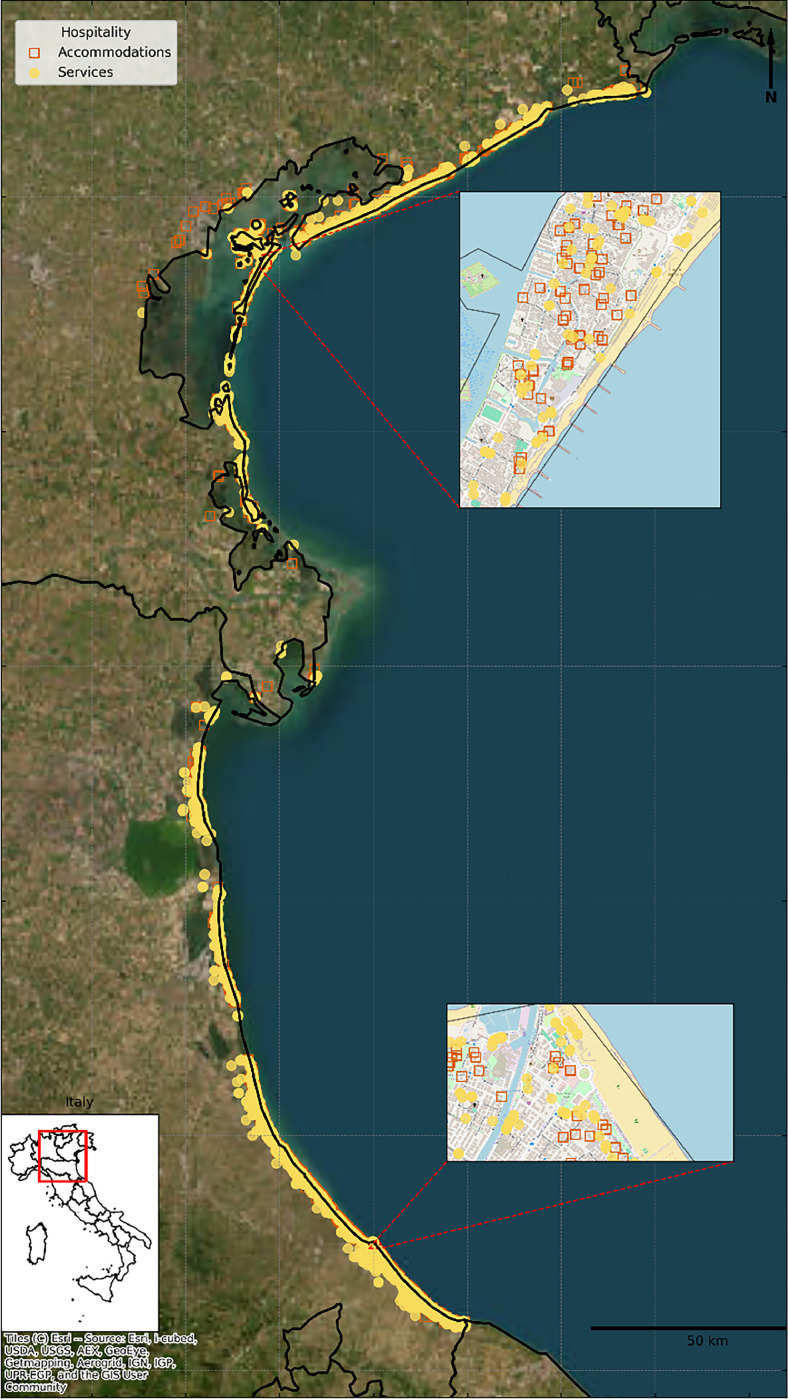
Spatial distribution of coastal hospitality establishments across Veneto and Emilia-Romagna regions, Northern Italy. The map displays the geographic coverage of the dataset, with 5030 accommodation establishments and 4992 service facilities represented by point locations along the Adriatic coastline. Two detailed insets highlight high-density hospitality clusters: the northern inset shows the Venice metropolitan area and Lido di Venezia, while the southern inset displays the Rimini-Riccione coastal corridor. Point locations are derived from multi-source data integration including web scraping, API queries, and crowdsourced repositories as detailed in the methodology.

Table 1 provides a structured classification of the variables available in the dataset, including their sources and formats and Fig. 1 illustrates the geographic overview of the dataset coverage and spatial distribution patterns of hospitality infrastructure.

**Table 1:** Structured classification of variables in the coastal hospitality dataset.

Category	Variables	Description	Unit/Format	Source	Links	Database
**Geospatial Variables**	Latitude, Longitude	Geographic coordinates defining spatial location	Decimal degrees (WGS84)	Google API Places/Eubucco/Overture Maps/Open Street Maps	https://developers.google.com/maps/documentation/places/web-service); https://eubucco.com/; https://overturemaps.org/;https://www.openstreetmap.org/	Accommodations and Services datasets
	Distance to sea	Euclidean distance to shoreline	meters	DEM Trinitaly	https://tinitaly.pi.ingv.it/	Accommodations and Services datasets
	Distance to waterways	Euclidean distance to nearest inland water body	meters	DEM Trinitaly	https://tinitaly.pi.ingv.it/	Accommodations and Services datasets
	Distance to estuaries	Euclidean distance to nearest river mouth	meters	DEM Trinitaly	https://tinitaly.pi.ingv.it/	Accommodations and Services datasets
	Current elevation	Height above mean sea level	meters	DEM Trinitaly	https://tinitaly.pi.ingv.it/	Accommodations and Services datasets
	Projected elevation (2030)	Elevation considering sea-level rise under RCP4.5 scenario	meters	Church et al., 2013	https://doi.org/10.1017/CBO9781107415324.026	Accommodations and Services datasets
**Physical Infrastructure**	Building area	Footprint of establishment structure	square meters (m²)	Eubucco/Overture Maps	https://eubucco.com/; https://overturemaps.org/	Accommodations and Services datasets
	Building height	Vertical extent of primary structure	meters	Eubucco/Overture Maps	https://eubucco.com/; https://overturemaps.org/	Accommodations and Services datasets
**Business Attributes**	Establishment type	Classification of hospitality facility	categorical (encoded)	Scraped/ Google API Places	https://developers.google.com/maps/documentation/places/web-service	Accommodations and Services datasets
	Capacity	Number of rooms/maximum occupancy	count	Scraped	Domains: booking.com, airbnb.com, tripadvisor.com, https://www.openstreetmap.org/	Accommodation dataset
	Price	Cost of accommodation/services (2023/2024)	Euros (€)	Scraped	Domains: booking.com, airbnb.com, tripadvisor.com, https://www.openstreetmap.org/	Accommodations and Services datasets
	Entry Price	Standard entry price (for applicable establishments) (2023/2024)	Euros (€)	Scraped	Domains: booking.com, airbnb.com, tripadvisor.com, https://www.openstreetmap.org/	Services dataset
	Customer rating	Satisfaction assessment from clientele	1–5 scale	Scraped	Domains: booking.com, airbnb.com, tripadvisor.com, https://www.openstreetmap.org/	Accommodations and Services datasets
**Environmental Features**	Beach access	Availability and type of shoreline access	categorical (encoded)	Scraped	Domains: booking.com, airbnb.com, tripadvisor.com, https://www.openstreetmap.org/	Accommodations and Services datasets
	Pool availability	Presence and type of swimming facilities	categorical (encoded)	Scraped	Domains: booking.com, airbnb.com, tripadvisor.com, https://www.openstreetmap.org/	Accommodations and Services datasets
	Green infrastructure	Presence of vegetation and natural elements	categorical (encoded)	Scraped	Domains: booking.com, airbnb.com, tripadvisor.com, https://www.openstreetmap.org/	Accommodations and Services datasets
	Environmental certification	Formal recognition of environmental certification	categorical (encoded)	Scraped	Domains: booking.com, airbnb.com, tripadvisor.com, https://www.openstreetmap.org/	Accommodations and Services datasets
	Sustainability Practices	Presence of sustainable practices	categorical (encoded)	Scraped	Domains: booking.com, airbnb.com, tripadvisor.com, https://www.openstreetmap.org/	Accommodation dataset

### 3.1 Dataset structure and organization

The coastal hospitality database is systematically organized into two complementary components that together provide comprehensive coverage of the hospitality ecosystem across the Northern Adriatic coastal regions (Fig. 1). The accommodations dataset (coastal_hospitality_accommodations.csv) captures the core lodging infrastructure, encompassing hotels, holiday rentals, bed and breakfasts, camping facilities, and guesthouses that form the primary hospitality backbone of the region. The services dataset (coastal_hospitality_services.csv) documents the supporting infrastructure, including restaurants, retail establishments, cultural venues, entertainment facilities, and seaside services that complement the accommodation sector and collectively define the hospitality experience.

This dual-dataset structure reflects the integrated nature of coastal hospitality operations, where accommodation establishments and service facilities function as interconnected components of the tourism system. The datasets share a common spatial framework and variable structure, enabling seamless integration for comprehensive vulnerability assessments while maintaining analytical flexibility for sector-specific investigations.

### 3.2 Variable relationships and interdependencies

The database variables are organized into four interconnected categories that capture the multidimensional nature of coastal hospitality vulnerability:

**Geospatial Variables** (latitude, longitude, distances, elevation) form the foundational layer for spatial analyses. Distance relationships to water bodies simultaneously determine attractiveness and vulnerability, while elevation variables combine with sea-level rise projections to create temporal vulnerability gradients. **Physical Infrastructure** characteristics (building area, height) interact with proximity metrics to define exposure profiles. Larger footprints in low-elevation coastal zones represent concentrated assets at risk, while building height can provide protection against inundation events. **Business Attributes** (establishment type, capacity, pricing, ratings) function as vulnerability modulators influencing adaptive capacity and economic resilience. Higher-rated establishments with premium pricing typically possess greater resources for adaptation measures. **Environmental Features** (beach access, green infrastructure, certifications) serve as risk modifiers that amplify or mitigate vulnerability. Beach access types relate directly to coastal exposure, while environmental certifications indicate proactive management that may enhance climate resilience.

### 3.3 Multi-scale analytical capabilities

The database structure supports analysis across multiple spatial and organizational scales. Establishment-level granularity enables property-specific vulnerability assessments, while categorical aggregations (by establishment type, size, or environmental features) support sector-wide policy analysis. Spatial clustering capabilities are enhanced through the integration of point-level coordinates with building footprints and proximity metrics, enabling the identification of vulnerability hotspots and risk concentration patterns.

## 4 Experimental Design, Materials and Methods

The methodological framework for developing this comprehensive hospitality sector database comprised four principal phases: (1) data acquisition from multiple sources, (2) spatial data integration and harmonization, (3) quality control and validation, and (4) attribute enrichment.

### 4.1 Data acquisition

The data acquisition process employed a multi-source approach to ensure comprehensive coverage of the hospitality sector across the coastal regions of Veneto and Emilia-Romagna. The systematic extraction protocol integrated automated web scraping, API queries, and crowdsourced data repositories.

### 4.2 Web scraping implementation

The web scraping component targeted primary booking platforms dominating the Italian coastal hospitality market, including booking.com, airbnb.com, and tripadvisor.com. A systematic grid-based search strategy was implemented using 500-meter radius centroids covering the entire study area (43.7°−45.7°N, 12.2°−13.8°E). The technical implementation utilized BeautifulSoup and Selenium libraries (Python language) within an automated browser environment.

The scraping process followed a structured protocol: First, Chrome WebDriver instances were initialized with rotating User-Agent headers to ensure request diversity. Cookie consent mechanisms were automated to maintain GDPR compliance throughout the data collection process. For each coordinate point in the search grid, the system navigated to the location, executed nearby establishment searches, and implemented scroll pagination to capture all available results, with a maximum of 10 scroll actions per search to respect server resources.

Data extraction employed specific CSS selectors and XPath queries to capture establishment attributes. Name fields were extracted using div.qBF1Pd.fontHeadlineSmall selectors, while geographic coordinates were parsed from URL parameters in the format !3d[latitude]!4d[longitude]. Price information underwent normalization to EUR values, and amenity details were processed as semi-colon delimited strings.

Rate limiting protocols were rigorously implemented to ensure ethical data collection. Request intervals followed a random uniform distribution between 3 and 10 s. Failed requests triggered an exponential backoff mechanism starting at 2 s (2^n seconds, maximum 5 retries). Daily extraction limits were set at 1000 establishments per session to prevent server overload. All scraping activities respected robots.txt directives and adhered to the terms of service of targeted platforms [10].

To address efficiency constraints, the data collection workflow was significantly enhanced through the implementation of visual web automation techniques, following established paradigms in end-user development [11] and visual data extraction frameworks [12]. The automated implementation streamlined several critical processes: browser session management with persistent authentication states, dynamic form filling for location-based searches, intelligent pagination handling with automatic scroll detection, and structured data extraction with built-in error handling. This methodological enhancement enabled more comprehensive data coverage while reducing the technical barriers associated with traditional web scraping approaches.

### 4.3 Google places API integration

Structured API queries complemented web scraping efforts by providing authoritative establishment data through Google Places API. The implementation employed comprehensive search parameters targeting hospitality-relevant establishment types including ‘lodging’, ‘restaurant’, ‘cafe’, ‘park’, ‘amusement_park’, ‘zoo’, and ‘museum’ within 500-meter radii of grid centroids [13].

The API query configuration requested specific data fields essential for vulnerability assessment: establishment names, geometric coordinates, customer ratings, price levels, formatted addresses, websites, unique place identifiers, establishment types, and total user ratings. This targeted approach maximized data utility while minimizing API quota consumption.

Rate limiting mechanisms ensured compliance with API usage policies. Query rates were restricted to 10 requests per second through programmatic delays. Daily quotas of 150,000 requests were monitored via internal tracking systems. Pagination requests implemented mandatory 2-second delays between next_page_token calls. Failed requests triggered a retry strategy with progressive delays of 2, 4, and 8 s across three attempts.

Response handling procedures addressed all potential API error states. OVER_QUERY_LIMIT errors initiated 24-hour pause periods before resumption. REQUEST_DENIED responses triggered credential validation routines. INVALID_REQUEST errors were logged and skipped to maintain processing continuity. ZERO_RESULTS responses marked areas as completely processed. All other errors invoked exponential backoff retry mechanisms.

Data filtering criteria ensured quality and relevance of extracted information. Geographic bounds validation confirmed coordinates fell within the study area. Establishment type filtering retained only hospitality-relevant venues. Place identifier deduplication eliminated redundant entries across overlapping searches. Completeness thresholds required minimum viable data fields for record retention. Rating validations ensured values fell within expected ranges (1.0–5.0) with non-zero user participation.

### 4.4 Crowdsourced data integration

Following systematic extraction from commercial platforms, the dataset incorporated open-source geographic data from OpenStreetMap [14], Eubucco [1], and Overture Maps [2]. These repositories provided building footprints, structural characteristics, and supplementary establishment attributes. Integration of crowdsourced data required additional validation procedures to address inherent quality variability ([15]; Antoniou, 2017).

### 4.5 Spatial data integration and harmonization

The integration of multiple data sources necessitated a robust spatial matching framework to identify duplicate entities and consolidate attribute information. The methodology employed a hierarchical matching approach with geographical location as the primary criterion and attribute similarity as secondary validation.

Establishments from different sources were matched using a 50-meter spatial proximity threshold implemented through QGIS spatial join operations and custom Python scripts [16]. Within this threshold, attribute similarity measures computed Levenshtein distances for establishment names, with matches confirmed at similarity scores exceeding 0.8. Type concordance provided additional validation, with establishment categories mapped to a unified classification schema.

Data precedence rules prioritized authoritative sources: Google Places API data superseded web-scraped information, which in turn took precedence over crowdsourced data. This hierarchy ensured optimal data quality while maintaining comprehensive coverage.

### 4.6 Quality control and validation

A multi-tiered quality control framework ensured data integrity throughout the collection and integration process:

***Automated Validation Procedures*:** All establishment coordinates underwent geometric validation to identify spatial anomalies. Coordinate bounds checking confirmed locations within valid ranges (−90° to 90° latitude, −180° to 180° longitude) and within the study area boundaries. Land-water verification using coastline shapefiles identified implausible marine positions for terrestrial establishments. ***Topological Consistency Assessment*:** Spatial relationships between establishments and coastal features underwent logical evaluation. The process identified and corrected anomalous configurations such as accommodation facilities positioned in water bodies or beach clubs located inland beyond reasonable distances. ***Statistical Outlier Detection*:** Spatial clustering algorithms identified establishments with anomalous locations relative to their categories. Price outliers underwent additional scrutiny, with values exceeding three standard deviations flagged for manual review. Establishment density hotspots were validated against known tourism concentrations. ***Cross-Source Validation*:** For establishments matched across multiple sources, attribute consistency metrics were computed. Discrepancies in critical fields (location, type, capacity) triggered manual reconciliation following established protocols [17]. ***Expert Review Process*:** A stratified random sample comprising 30 % of integrated records underwent manual validation by domain experts. Establishment details were verified against authoritative sources, current satellite imagery, and street-level photography. The validation achieved 85 % concordance with automated classifications, exceeding accepted thresholds for spatial databases. Systematic quality metrics tracking enabled continuous improvement throughout the data development process (Herzog et al., 2007).

### 4.7 Attribute enrichment

The validated dataset underwent enrichment with attributes critical for climate vulnerability assessment. Elevation data from the TINITALY digital terrain model [18] provided 10-meter resolution height values for each establishment location. Distance calculations to coastal features (sea, waterways, estuaries) employed Euclidean metrics computed through spatial analysis routines.

Sea-level rise projections integrated IPCC-AR5 ensemble model outputs [19,20]. The ensemble comprised 20 global models at 1° spatial resolution, with 12 models providing Mediterranean-specific projections. Ensemble median values under RCP 4.5 scenarios were spatially interpolated to establishment locations. The analytical framework employed a bathtub inundation model [21], subtracting projected 2030 sea levels from current elevations to assess exposure risk.

Environmental feature classification required manual interpretation supplemented by automated text analysis. Establishment descriptions underwent natural language processing to identify sustainability certifications, green infrastructure presence, and beach access types. Binary encoding captured feature presence/absence, while categorical variables employed hierarchical classification schemes.

The resultant integrated dataset provides unprecedented spatial detail on hospitality infrastructure across coastal Veneto and Emilia-Romagna, enabling sophisticated vulnerability assessments for climate adaptation planning.

## Limitations

While this dataset represents a comprehensive effort to document the coastal hospitality sector in Veneto and Emilia-Romagna, several limitations warrant consideration. The inherent subjectivity in human labeling processes during validation may introduce inconsistencies despite the implementation of standardized protocols [22]. Temporal aspects of data collection also impact dataset currency, as urban development and land use changes can rapidly alter the accuracy of the collected information.

Although integration challenges between diverse data sources have been systematically addressed [23], the dynamic nature of geographic features necessitates ongoing validation and updates. The integration of OpenStreetMap and Overture Maps data introduced variability due to their crowdsourced nature (Kaur et al., 2017; Antoniou, 2017) and technical constraints [24]. In particular, establishment classification schemes exhibited some inconsistency between different data sources, requiring manual harmonization.

Additionally, while building footprints and elevations were derived from the most current available data, local modifications such as flood protection measures at individual establishments could not be comprehensively documented. The sea-level rise projections utilized in this dataset, derived from the IPCC-AR5 2015 ensemble [19,20], represent the scientific consensus available at the time of data compilation. However, recent studies suggest that these projections may underestimate regional sea-level rise variability due to the complex dynamics of the Northern Adriatic, including localized climatic interactions and wind pattern changes [[25], [26], [27]]. Consequently, the vulnerability assessments based on these projections should be interpreted as conservative estimates of minimum risk scenarios rather than comprehensive representations of potential future impacts. Therefore, inherent uncertainties in climate modelling may affect the accuracy of vulnerability assessments based on these projections [19,20].

These limitations notwithstanding, this multi-source approach enabled the construction of the most comprehensive hospitality sector database currently available for these coastal regions, forming a robust foundation for spatial vulnerability analysis and adaptation planning.

## Ethics Statement

The authors have read and follow the ethical requirements for publication in Data in Brief and confirm that the current work does not involve human subjects, animal experiments, or any data collected from social media platforms. All data collection activities adhered to the European General Data Protection Regulation (GDPR) guidelines. Web scraping procedures followed established ethical protocols, respecting robots.txt directives and implementing rate-limiting measures to prevent server overloading. Commercial data usage complies with licensing agreements, and OpenStreetMap data has been utilized in accordance with its open data license requirements.

## CRediT authorship contribution statement

**Vilane Gonçalves Sales:** Conceptualization, Methodology, Software, Validation, Data curation, Formal analysis, Writing – review & editing.

## Data Availability

ZenodoA Coastal Hospitality Sector Database for Vulnerability Assessments in Veneto and Emilia-Romagna, Italy (Original data).
